# Supplementary material for: Regulation and Molecular Mechanism of TLR5 on Resistance to Escherichia coli F18 in Weaned Piglets
Source: Animals (Basel). 2019 Sep 27;9(10):735. doi: 10.3390/ani9100735 (PMC6827021; doi:10.3390/ani9100735)
Supplement: Supplementary file 1 [file animals-09-00735-s001.zip › Table S2.doc]

**Table S2** FPKM information

| transcript_id | gene_id | gene_name | length | K_FPKM | M_FPKM | log2(foldchange) | p-value | q-value |
| --- | --- | --- | --- | --- | --- | --- | --- | --- |
| XM_005660361.1 | 100157179 | ZNF618 | 2868 | 0.000355572 | 0.679208 | -10.8995 | 0.04205 | 0.751804 |
| XM_005654962.1 | 100521252 | CLK4 | 1848 | 0.00190929 | 1.16537 | -9.25354 | 0.03525 | 0.698207 |
| XM_005661091.1 | 733603 | LOC733603 | 677 | 2.43804 | 57.0648 | -4.54881 | 5.00E-05 | 0.00737594 |
| XM_005665062.1 | 100738064 | PLA2G2A | 1106 | 0.087532 | 1.46744 | -4.06734 | 0.0218 | 0.552653 |
| XM_005658975.1 | 100517370 | LOC100517370 | 1250 | 0.35504 | 3.59379 | -3.33945 | 5.00E-05 | 0.00737594 |
| XM_005652369.1 | 397585 | FETUIN | 1587 | 0.198817 | 1.99314 | -3.32553 | 0.00195 | 0.126847 |
| XM_005655649.1 | 102167487 | LOC102167487 | 3594 | 0.125172 | 1.02777 | -3.03753 | 0.0002 | 0.0235047 |
| XM_003482017.2 | 100739434 | LOC100739434 | 2010 | 0.0689632 | 0.539957 | -2.96895 | 0.01145 | 0.389665 |
| XM_005667215.1 | 100623257 | LOC100623257 | 1564 | 0.308764 | 2.30164 | -2.89809 | 0.0005 | 0.0481982 |
| XM_003483700.2 | 100737264 | LOC100737264 | 3499 | 0.130384 | 0.963218 | -2.8851 | 0.0014 | 0.101095 |
| XM_005655794.1 | 102167543 | LOC102167543 | 1333 | 0.43838 | 3.2242 | -2.87869 | 0.00025 | 0.0279817 |
| XM_003481193.1 | 100737346 | LOC100737346 | 818 | 10.8793 | 76.6756 | -2.81719 | 5.00E-05 | 0.00737594 |
| XM_005664953.1 | 100736849 | LOC100736849 | 1690 | 2.43512 | 16.3114 | -2.74382 | 5.00E-05 | 0.00737594 |
| XM_005662708.1 | 102166849 | LOC102166849 | 763 | 2.56298 | 16.7768 | -2.71058 | 5.00E-05 | 0.00737594 |
| XM_005658352.1 | 100625274 | LOC100625274 | 1682 | 0.103706 | 0.666868 | -2.6849 | 0.01505 | 0.456445 |
| XM_003133815.4 | 100513317 | LOC100513317 | 812 | 0.178059 | 1.13425 | -2.67131 | 0.0244 | 0.586215 |
| XM_003359590.2 | 100512076 | SLC40A1 | 3499 | 0.16303 | 1.01203 | -2.63403 | 0.00075 | 0.0641814 |
| XM_003123279.2 | 100524668 | LOC100524668 | 1296 | 0.105482 | 0.614227 | -2.54178 | 0.04765 | 0.791145 |
| XM_005655795.1 | 102167650 | LOC102167650 | 1245 | 0.741664 | 4.01643 | -2.43708 | 5.00E-05 | 0.00737594 |
| XM_005663709.1 | 100523492 | GBP5 | 2414 | 5.23168 | 27.5818 | -2.39837 | 5.00E-05 | 0.00737594 |
| XM_005662795.1 | 100518658 | LOC100518658 | 926 | 1.58366 | 7.91931 | -2.32212 | 5.00E-05 | 0.00737594 |
| XM_003362105.2 | 100624393 | LOC100624393 | 1718 | 0.322416 | 1.46883 | -2.18768 | 0.001 | 0.0796319 |
| NM_001243873.1 | 100511639 | LOC100511639 | 1370 | 0.379183 | 1.62139 | -2.09626 | 0.00855 | 0.331261 |
| XM_005655796.1 | 102167769 | LOC102167769 | 694 | 1.77606 | 7.44394 | -2.06739 | 0.0007 | 0.0610889 |
| XM_003361626.1 | 100621708 | LOC100621708 | 3423 | 0.802149 | 3.35304 | -2.06353 | 5.00E-05 | 0.00737594 |
| XM_005658540.1 | 102161157 | LOC102161157 | 417 | 5.40437 | 22.0202 | -2.02663 | 0.0001 | 0.0134312 |
| NM_001114289.2 | 100135681 | CXCL9 | 450 | 16.9458 | 62.6021 | -1.88528 | 5.00E-05 | 0.00737594 |
| XM_005657677.1 | 102157588 | LOC102157588 | 1118 | 1.33807 | 4.67574 | -1.80504 | 0.00195 | 0.126847 |
| XM_003484284.1 | 100737466 | LOC100737466 | 1874 | 1.70838 | 5.79123 | -1.76125 | 0.00055 | 0.0514701 |
| XM_005666529.1 | 100624787 | LOC100624787 | 879 | 0.294307 | 0.994354 | -1.75644 | 0.03985 | 0.737238 |
| XM_005661808.1 | 102165673 | LOC102165673 | 2296 | 0.320648 | 1.07473 | -1.74491 | 0.01465 | 0.449241 |
| NM_213931.1 | 396971 | ALOX12 | 2901 | 0.260726 | 0.842417 | -1.692 | 0.0033 | 0.181016 |
| XM_005666806.1 | 100626178 | FGA | 2123 | 0.23631 | 0.751095 | -1.66832 | 0.0301 | 0.647984 |
| XM_003122320.4 | 100521600 | LOC100521600 | 3234 | 0.406661 | 1.28656 | -1.66162 | 0.0056 | 0.253696 |
| XM_005655042.1 | 100627004 | LOC100627004 | 1600 | 6.69159 | 21.1462 | -1.65997 | 0.0297 | 0.643894 |
| XM_005657679.1 | 102158041 | LOC102158041 | 1348 | 1.17447 | 3.64774 | -1.63499 | 0.0447 | 0.770466 |
| NM_213876.1 | 396900 | AMCF-II | 1489 | 1.72832 | 5.36132 | -1.63322 | 0.00045 | 0.0443175 |
| XM_005668752.1 | 100512433 | PYY | 801 | 12.128 | 37.556 | -1.6307 | 0.00175 | 0.117579 |
| NM_213847.1 | 396845 | ICA | 2315 | 0.530785 | 1.63271 | -1.62107 | 0.00325 | 0.17925 |
| NM_001244733.1 | 100512997 | INMT | 969 | 1.09427 | 3.32982 | -1.60548 | 0.00465 | 0.225277 |
| NM_214069.1 | 397139 | FUT2 | 2316 | 2.53466 | 7.68435 | -1.60013 | 0.00015 | 0.0185889 |
| XM_005666877.1 | 102159296 | LOC102159296 | 4150 | 0.885413 | 2.64815 | -1.58056 | 0.0003 | 0.0322719 |
| XM_005672404.1 | 100620294 | LOC100620294 | 1772 | 0.511608 | 1.516 | -1.56716 | 0.0167 | 0.48321 |
| XM_003129240.4 | 100513297 | LOC100513297 | 687 | 1.02358 | 2.90736 | -1.50609 | 0.02375 | 0.577685 |
| XM_005657681.1 | 100620484 | LOC100620484 | 1625 | 1.18614 | 3.35035 | -1.49804 | 0.00495 | 0.234022 |
| XM_003123094.4 | 100513844 | FUT3 | 1421 | 5.82456 | 16.3322 | -1.4875 | 0.0008 | 0.0674775 |
| NM_214020.1 | 397083 | EGF | 305 | 5.18926 | 14.2266 | -1.45499 | 0.01095 | 0.380922 |
| XM_003483081.1 | 100738021 | LOC100738021 | 930 | 0.732303 | 2.00632 | -1.45404 | 0.0276 | 0.623178 |
| XM_003127418.4 | 100522142 | LOC100522142 | 1979 | 1.14001 | 3.02485 | -1.40782 | 0.00085 | 0.070722 |
| XM_003128317.4 | 100512544 | LOC100512544 | 937 | 3.59164 | 9.32769 | -1.37688 | 0.00095 | 0.0767774 |
| XM_005658532.1 | 100627397 | LOC100627397 | 2126 | 2.1569 | 5.58058 | -1.37145 | 0.00185 | 0.122145 |
| XM_005661568.1 | 102165335 | LOC102165335 | 3099 | 0.243189 | 0.619746 | -1.3496 | 0.01655 | 0.480546 |
| XM_001924902.3 | 100153042 | LOC100153042 | 914 | 0.702818 | 1.7234 | -1.29404 | 0.0423 | 0.753503 |
| XM_003134585.4 | 100523477 | GIMAP2 | 1382 | 0.686306 | 1.67274 | -1.28529 | 0.0179 | 0.500179 |
| XM_005673835.1 | 100524357 | ZCCHC16 | 1802 | 0.583305 | 1.41778 | -1.28131 | 0.01385 | 0.435796 |
| XM_003355059.3 | 100152118 | OPRK1 | 2116 | 0.256577 | 0.621525 | -1.27642 | 0.048 | 0.793686 |
| XM_005658731.1 | 102168053 | LOC102168053 | 396 | 8.95671 | 21.6856 | -1.2757 | 0.0056 | 0.253696 |
| NM_001123196.1 | 493186 | SOCS3 | 590 | 2.33904 | 5.63461 | -1.2684 | 0.01345 | 0.427502 |
| XM_005664534.1 | 100524089 | LOC100524089 | 2899 | 0.323946 | 0.779242 | -1.26632 | 0.02435 | 0.585513 |
| NM_001206402.1 | 100154124 | TRIM31 | 2055 | 12.4238 | 29.6212 | -1.25352 | 0.0002 | 0.0235047 |
| NM_214055.1 | 397122 | IL1B | 1460 | 0.567332 | 1.3358 | -1.23544 | 0.0291 | 0.637848 |
| XM_005672371.1 | 100514433 | NUTM2G | 2417 | 0.456934 | 1.06075 | -1.21502 | 0.03435 | 0.691551 |
| XM_005655072.1 | 100522669 | LOC100522669 | 1471 | 2.33063 | 5.36018 | -1.20156 | 0.00585 | 0.26108 |
| XM_005667980.1 | 100626755 | LOC100626755 | 1816 | 0.450037 | 1.03298 | -1.1987 | 0.04825 | 0.795661 |
| XM_003482496.2 | 100739437 | LOC100739437 | 1704 | 1.69863 | 3.8928 | -1.19644 | 0.02295 | 0.567326 |
| XM_005653616.1 | 102162205 | LOC102162205 | 5474 | 0.39695 | 0.898875 | -1.17916 | 0.0045 | 0.220739 |
| XM_005663244.1 | 100152036 | LOC100152036 | 1134 | 1.91688 | 4.27018 | -1.15553 | 0.03285 | 0.677702 |
| XM_003123754.4 | 100513005 | THBS4 | 2984 | 0.55302 | 1.22669 | -1.14936 | 0.0231 | 0.569238 |
| XM_005665757.1 | 102166052 | LOC102166052 | 1584 | 0.706632 | 1.56515 | -1.14727 | 0.0355 | 0.700209 |
| NM_001244695.1 | 100512662 | PLA2G2D | 1239 | 4.05333 | 8.88667 | -1.13254 | 0.00325 | 0.17925 |
| XM_005656792.1 | 102167736 | LOC102167736 | 1163 | 4.50078 | 9.58474 | -1.09057 | 0.0131 | 0.421027 |
| NM_001206441.1 | 733650 | TAP2 | 2617 | 1.84134 | 3.91261 | -1.08738 | 0.0028 | 0.162234 |
| XM_005663706.1 | 100523310 | GBP6 | 2539 | 10.1847 | 21.3527 | -1.06801 | 0.0004 | 0.0403802 |
| NM_214317.1 | 397586 | AFP | 2016 | 3.96863 | 8.25713 | -1.057 | 0.00715 | 0.295357 |
| XM_005658277.1 | 100623336 | LOC100623336 | 2673 | 1.65624 | 3.41633 | -1.04453 | 0.0036 | 0.191802 |
| XM_005663708.1 | 102161784 | LOC102161784 | 1485 | 1.87667 | 3.81889 | -1.02498 | 0.0106 | 0.372606 |
| XM_005652468.1 | 102160446 | LOC102160446 | 1847 | 2.58814 | 5.2594 | -1.02299 | 0.0128 | 0.416031 |
| XM_005666775.1 | 100169744 | CXCL11 | 1063 | 2.72821 | 5.54031 | -1.02201 | 0.0195 | 0.522624 |
| XM_003125922.4 | 100523668 | LOC100523668 | 1772 | 25.3937 | 50.9355 | -1.0042 | 0.00125 | 0.0935695 |
| XM_005662125.1 | 100307052 | SOCS1 | 1250 | 2.9094 | 5.68288 | -0.965903 | 0.0349 | 0.694984 |
| XM_003126503.2 | 100523789 | LOC100523789 | 1249 | 2.43991 | 4.63711 | -0.926401 | 0.0257 | 0.601663 |
| XM_005661785.1 | 100739218 | LOC100739218 | 611 | 30.8698 | 58.2849 | -0.916926 | 0.04425 | 0.767777 |
| XM_003123974.4 | 100520726 | EGR1 | 3165 | 4.49925 | 8.38359 | -0.897884 | 0.0075 | 0.305079 |
| XM_003356781.3 | 100627413 | ASB2 | 1834 | 0.967249 | 1.79728 | -0.893856 | 0.04945 | 0.804271 |
| XM_003481515.2 | 100737841 | LOC100737841 | 2351 | 13.6479 | 25.3249 | -0.891875 | 0.00245 | 0.149059 |
| XM_005663716.1 | 100155195 | GBP7 | 2615 | 43.9195 | 80.3356 | -0.871177 | 0.0049 | 0.232594 |
| XM_005653499.1 | 100736850 | LOC100736850 | 946 | 5.80542 | 10.509 | -0.856151 | 0.02175 | 0.55208 |
| XM_003133981.3 | 397251 | PLK2 | 2801 | 5.01494 | 9.01548 | -0.846173 | 0.00785 | 0.314241 |
| XM_003482521.2 | 100512797 | GVIN1 | 6558 | 35.2187 | 63.2266 | -0.84419 | 0.01515 | 0.457511 |
| XM_001927551.2 | 100154770 | IER3 | 1646 | 1.99319 | 3.55114 | -0.833206 | 0.0294 | 0.640442 |
| NM_001143710.1 | 100233184 | GZMB | 890 | 32.8899 | 58.142 | -0.821935 | 0.01615 | 0.474303 |
| XM_003361092.2 | 100620361 | LOC100620361 | 1571 | 12.1985 | 21.3648 | -0.808533 | 0.01055 | 0.37159 |
| XM_005654299.1 | 102158723 | LOC102158723 | 1344 | 2.31495 | 4.05279 | -0.807939 | 0.0354 | 0.699312 |
| XM_005671902.1 | 397689 | XIRP2 | 12126 | 0.332135 | 0.568779 | -0.776098 | 0.032 | 0.669172 |
| NM_213776.1 | 396662 | CD2 | 1023 | 30.5131 | 51.835 | -0.764499 | 0.0072 | 0.296814 |
| XM_003124235.3 | 100519082 | LOC100519082 | 1598 | 18.0849 | 30.4681 | -0.752511 | 0.02025 | 0.531909 |
| XM_003131994.2 | 396711 | MYHC | 6080 | 0.750228 | 1.26058 | -0.748689 | 0.03325 | 0.680874 |
| NM_001198926.1 | 100526762 | GZMA | 799 | 10.2133 | 16.9964 | -0.734787 | 0.0358 | 0.702393 |
| XM_005670219.1 | 102161685 | LOC102161685 | 7406 | 25.0494 | 41.5199 | -0.729026 | 0.04775 | 0.79178 |
| NM_001001265.1 | 397406 | FCER1G | 579 | 12.2503 | 20.28 | -0.727245 | 0.03775 | 0.718851 |
| XM_003482441.2 | 100518025 | SCLT1 | 1725 | 2.56295 | 4.23645 | -0.725047 | 0.0497 | 0.806175 |
| NM_001123202.1 | 100144476 | TLR5 | 2572 | 4.7384 | 7.83192 | -0.724966 | 0.01525 | 0.45886 |
| XM_003126977.2 | 100517894 | LPCAT2 | 3375 | 3.34616 | 5.49759 | -0.716295 | 0.0281 | 0.629229 |
| XM_003130821.4 | 100515572 | LOC100515572 | 986 | 5.84649 | 9.54045 | -0.706487 | 0.0467 | 0.78517 |
| XM_005653274.1 | 100525924 | LOC100525924 | 2731 | 2.51913 | 4.10592 | -0.704783 | 0.03465 | 0.693433 |
| NM_001097478.1 | 100037994 | RPL14 | 710 | 441.718 | 719.568 | -0.704006 | 0.033 | 0.678909 |
| XM_003130633.4 | 100515919 | LOC100515919 | 1371 | 61.9041 | 99.9835 | -0.691656 | 0.0128 | 0.416031 |
| XM_005662121.1 | 100738647 | LOC100738647 | 2141 | 3.46659 | 5.57916 | -0.686531 | 0.0349 | 0.694984 |
| NM_001123113.1 | 100144486 | FOS | 1143 | 30.9725 | 49.2059 | -0.667846 | 0.0213 | 0.546064 |
| XM_005669122.1 | 100519919 | ITGAE | 3493 | 10.8773 | 17.2568 | -0.665835 | 0.0405 | 0.741674 |
| NM_214227.1 | 397455 | CD3E | 1232 | 22.6845 | 35.9864 | -0.665745 | 0.02655 | 0.611313 |
| XM_001926804.3 | 497623 | P2RX7 | 2612 | 12.0476 | 18.9856 | -0.656161 | 0.02845 | 0.631651 |
| XM_005658955.1 | 102163208 | APOBR | 2269 | 3.04565 | 4.79449 | -0.654628 | 0.04535 | 0.775133 |
| NM_214412.1 | 403103 | CYP1A1 | 2860 | 98.6636 | 155.248 | -0.653987 | 0.0472 | 0.788034 |
| XM_005656775.1 | 100525885 | LOC100525885 | 3349 | 7.21699 | 11.2872 | -0.645224 | 0.0311 | 0.659445 |
| NM_001008691.1 | 494019 | CXCL10 | 1129 | 52.7812 | 81.4893 | -0.626586 | 0.03235 | 0.672998 |
| XM_005668209.1 | 100516289 | LOC100516289 | 1872 | 7.76984 | 11.9353 | -0.619273 | 0.03885 | 0.729067 |
| XM_005671469.1 | 100157959 | AFAP1L2 | 3667 | 3.7138 | 5.65681 | -0.607091 | 0.04865 | 0.797885 |
| XM_005667971.1 | 100517362 | LOC100517362 | 6178 | 2.58824 | 3.93926 | -0.605954 | 0.04175 | 0.749481 |
| XM_003483035.2 | 100737474 | LOC100737474 | 3014 | 4.48405 | 6.81182 | -0.603238 | 0.0437 | 0.763399 |
| XM_005658662.1 | 102167956 | LOC102167956 | 612 | 44.1056 | 66.4869 | -0.592107 | 0.03575 | 0.701998 |
| XM_003361556.2 | 100625876 | SLC2A5 | 1290 | 24.1815 | 35.8181 | -0.566786 | 0.0444 | 0.768771 |
| XM_005671857.1 | 100522709 | UPP2 | 2038 | 21.3467 | 31.3177 | -0.552963 | 0.04955 | 0.805062 |
| XM_005654912.1 | 102161910 | LOC102161910 | 273 | 0 | 2.46787 | #NAME? | 0.0001 | 0.0134312 |
| NM_213856.2 | 396861 | LCN1 | 172 | 0 | 14.0398 | #NAME? | 0.00035 | 0.0363207 |
| NM_001244642.1 | 100156741 | LOC100156741 | 411 | 0 | 14.3293 | #NAME? | 5.00E-05 | 0.00737594 |
| XM_005654402.1 | 574059 | EEF1A1 | 2029 | 3.97559 | 3.63219 | 0.130332 | 0.0432 | 0.76098 |
| XM_003127961.4 | 100524940 | LOC100524940 | 1913 | 100.936 | 66.7658 | 0.596258 | 0.0457 | 0.778473 |
| XM_001924181.4 | 100156093 | TUSC3 | 1201 | 29.549 | 19.016 | 0.635896 | 0.03195 | 0.66892 |
| XM_003357925.2 | 100158243 | SLC16A3 | 2162 | 15.7917 | 9.99216 | 0.660299 | 0.02825 | 0.630386 |
| XM_003124230.2 | 100127358 | IFITM1 | 780 | 167.891 | 105.121 | 0.675474 | 0.0289 | 0.635836 |
| XM_003121158.2 | 100156984 | ZC3H12D | 2790 | 7.19908 | 4.47499 | 0.685929 | 0.03455 | 0.693005 |
| XM_005654355.1 | 100516167 | SYNJ2 | 5842 | 3.97704 | 2.42921 | 0.71121 | 0.0178 | 0.499065 |
| XM_005659188.1 | 100628107 | LOC100628107 | 2146 | 6.04788 | 3.68807 | 0.713564 | 0.03875 | 0.727771 |
| NM_214423.1 | 403324 | CYP3A29 | 536 | 377.517 | 229.202 | 0.719926 | 0.03355 | 0.684236 |
| XM_003484045.2 | 100738822 | RP9 | 1302 | 5.95167 | 3.59858 | 0.725866 | 0.04615 | 0.780763 |
| NM_001134824.1 | 100126281 | CYP3A46 | 694 | 97.9735 | 59.2307 | 0.726046 | 0.01655 | 0.480546 |
| NM_001244717.1 | 100512737 | SLC13A2 | 2433 | 77.6243 | 46.4879 | 0.739653 | 0.01255 | 0.411127 |
| XM_005655241.1 | 102164809 | ABCG5 | 459 | 29.5395 | 17.5388 | 0.752098 | 0.03175 | 0.666811 |
| XM_003128013.4 | 100522082 | SLC5A9 | 1840 | 5.1125 | 3.02816 | 0.755588 | 0.0288 | 0.634428 |
| XM_003480608.2 | 100125551 | SLC2A8 | 1976 | 9.34517 | 5.53247 | 0.756299 | 0.0203 | 0.53233 |
| XM_005657599.1 | 100627765 | BOK | 999 | 6.15219 | 3.62878 | 0.761617 | 0.0461 | 0.780667 |
| XM_005663979.1 | 100154962 | PTPRR | 2699 | 8.92515 | 5.2297 | 0.771148 | 0.015 | 0.455419 |
| XM_005667372.1 | 406187 | APOC3 | 574 | 270.311 | 158.108 | 0.773712 | 0.03415 | 0.689591 |
| XM_001925115.2 | 100154016 | SH2D7 | 1863 | 2.89627 | 1.68894 | 0.778078 | 0.0418 | 0.74971 |
| XM_003361314.3 | 100621671 | LOC100621671 | 2196 | 5.30278 | 3.07632 | 0.785541 | 0.0264 | 0.609751 |
| XM_005666261.1 | 102167451 | LOC102167451 | 1038 | 11.6929 | 6.74314 | 0.794144 | 0.02015 | 0.530864 |
| NM_001115136.1 | 767625 | GPX2 | 799 | 17.8391 | 10.2406 | 0.800745 | 0.0288 | 0.634428 |
| XM_001927885.4 | 100154502 | PRAP1 | 603 | 159.722 | 91.4942 | 0.803807 | 0.0054 | 0.248548 |
| XM_005653435.1 | 100525112 | LOC100525112 | 1481 | 109.374 | 62.5094 | 0.807121 | 0.00735 | 0.301237 |
| XM_005667571.1 | 100514723 | CR1 | 4119 | 1.32975 | 0.751463 | 0.823383 | 0.0375 | 0.716703 |
| XM_003128394.2 | 100521201 | PGC | 1523 | 13.7929 | 7.78371 | 0.825399 | 0.0095 | 0.351184 |
| NM_214420.1 | 403215 | CYP2C49 | 1958 | 144.155 | 80.876 | 0.83384 | 0.03645 | 0.707372 |
| XM_005670013.1 | 100525008 | CCDC39 | 4118 | 3.83081 | 2.13374 | 0.844265 | 0.02 | 0.529484 |
| NM_214108.1 | 397196 | DPEP1 | 1462 | 24.3867 | 13.467 | 0.856665 | 0.0061 | 0.268164 |
| XM_005658511.1 | 100623504 | LOC100623504 | 1095 | 42.1091 | 23.1499 | 0.863126 | 0.01535 | 0.460689 |
| XM_003134735.2 | 100511294 | PTPRZ1 | 8006 | 0.94662 | 0.520181 | 0.863771 | 0.01895 | 0.514929 |
| XM_003123638.4 | 100518539 | GFPT2 | 3083 | 3.62906 | 1.98449 | 0.870829 | 0.0106 | 0.372606 |
| NM_001244385.1 | 100515954 | CDA | 501 | 35.247 | 19.0197 | 0.890004 | 0.0099 | 0.358178 |
| XM_001926306.5 | 100156526 | LOC100156526 | 2308 | 5.29846 | 2.85629 | 0.89143 | 0.00855 | 0.331261 |
| XM_005652669.1 | 397246 | VNN1 | 2977 | 11.9417 | 6.3646 | 0.907864 | 0.0398 | 0.73699 |
| NM_001097450.1 | 100037953 | C8A | 1901 | 4.57138 | 2.42193 | 0.916473 | 0.0186 | 0.510434 |
| XM_003130159.3 | 100523985 | DYNC1I1 | 2231 | 1.33527 | 0.697488 | 0.936887 | 0.0446 | 0.770154 |
| NM_214422.1 | 403322 | CYP3A39 | 299 | 183.56 | 95.4718 | 0.943102 | 0.0056 | 0.253696 |
| NM_001160080.1 | 100294675 | DGAT2 | 336 | 38.0486 | 19.7141 | 0.948614 | 0.0179 | 0.500179 |
| NM_001244884.1 | 100513890 | AGXT2 | 2185 | 2.56662 | 1.32665 | 0.952083 | 0.02865 | 0.632903 |
| XM_003125609.4 | 100524984 | MYBL1 | 4964 | 2.22025 | 1.14425 | 0.956325 | 0.00665 | 0.282396 |
| NM_001243483.1 | 100627480 | TFF3 | 784 | 370.337 | 189.721 | 0.96496 | 0.00245 | 0.149059 |
| NM_213852.1 | 396852 | MSMB | 512 | 45.4659 | 23.0773 | 0.978307 | 0.0037 | 0.19514 |
| XM_003359855.2 | 641346 | SLC6A19 | 3383 | 43.4549 | 21.8991 | 0.988648 | 0.0034 | 0.184563 |
| NM_001243221.1 | 100316854 | PSPH | 1397 | 8.37382 | 4.20023 | 0.995417 | 0.00495 | 0.234022 |
| NM_214110.1 | 397198 | SFTPD | 1347 | 20.1969 | 10.0899 | 1.00122 | 0.01015 | 0.363677 |
| NM_001038004.1 | 654325 | MMP9 | 2302 | 4.41083 | 2.18953 | 1.01043 | 0.0062 | 0.270536 |
| NM_001278765.1 | 100517472 | FFAR2-L | 1105 | 5.65667 | 2.79977 | 1.01465 | 0.01115 | 0.384559 |
| XM_005656187.1 | 100739514 | LOC100739514 | 1920 | 2.28169 | 1.12722 | 1.01733 | 0.04625 | 0.781705 |
| XM_005655986.1 | 100624036 | LOC100624036 | 1205 | 59.3301 | 29.1382 | 1.02585 | 0.0017 | 0.115319 |
| NM_001195399.1 | 100157103 | BMP2 | 1742 | 2.15759 | 1.05705 | 1.02939 | 0.0229 | 0.566984 |
| XM_003130757.4 | 100515334 | MYO3A | 5549 | 1.46857 | 0.719026 | 1.03029 | 0.0063 | 0.273125 |
| XM_001928587.2 | 100156557 | LOC100156557 | 624 | 64.2823 | 31.0287 | 1.05082 | 0.0012 | 0.0910347 |
| NM_214416.1 | 403124 | IYD | 658 | 9.49833 | 4.54294 | 1.06405 | 0.01325 | 0.423916 |
| XM_005668494.1 | 100518213 | LOC100518213 | 2789 | 46.8751 | 22.4118 | 1.06456 | 0.0012 | 0.0910347 |
| XM_003481432.2 | 100738802 | BCAN | 3328 | 1.54739 | 0.739129 | 1.06594 | 0.0191 | 0.516814 |
| NM_213999.2 | 397060 | DUOX2 | 5712 | 6.43476 | 3.05894 | 1.07285 | 0.0003 | 0.0322719 |
| XM_001927980.4 | 100154496 | TRIM31 | 2058 | 24.8263 | 11.7911 | 1.07417 | 0.00755 | 0.305878 |
| NM_001243912.1 | 100511898 | CD79B | 1245 | 11.111 | 5.2691 | 1.07636 | 0.0034 | 0.184563 |
| NM_001123127.1 | 396906 | HSP70 | 293 | 51.9633 | 24.4701 | 1.08647 | 0.00955 | 0.352017 |
| XM_003357400.2 | 100626414 | RAB7L1 | 2929 | 5.02928 | 2.36392 | 1.08917 | 0.03875 | 0.727771 |
| XM_005669094.1 | 100624611 | LOC100624611 | 2948 | 25.4045 | 11.9116 | 1.09271 | 0.0001 | 0.0134312 |
| NM_001123158.1 | 100144531 | PCK1 | 2591 | 45.5241 | 21.3158 | 1.09471 | 0.04365 | 0.762809 |
| XM_005658535.1 | 100627583 | LOC100627583 | 644 | 6.04543 | 2.81647 | 1.10196 | 0.03035 | 0.651179 |
| NM_001243855.1 | 100511551 | S1PR2 | 1778 | 4.37755 | 2.03923 | 1.1021 | 0.0172 | 0.489839 |
| XM_003359055.2 | 100623761 | BLK | 2097 | 1.0351 | 0.472385 | 1.13174 | 0.03385 | 0.687372 |
| XM_005668495.1 | 102167556 | LOC102167556 | 1573 | 34.1757 | 15.5252 | 1.13836 | 0.00075 | 0.0641814 |
| XM_005674257.1 | 100737314 | LOC100737314 | 2169 | 1.84132 | 0.83642 | 1.13844 | 0.03265 | 0.67585 |
| XM_005672303.1 | 100739320 | LOC100739320 | 1053 | 8.4131 | 3.80452 | 1.14492 | 0.0044 | 0.21788 |
| XM_003358448.2 | 100156039 | SLC38A3 | 2445 | 0.929816 | 0.39198 | 1.24617 | 0.02435 | 0.585513 |
| NM_214442.2 | 404699 | PBD-2 | 347 | 12.5913 | 5.24802 | 1.26258 | 0.0271 | 0.616628 |
| XM_005673043.1 | 100144531 | PCK1 | 2578 | 54.0095 | 22.4979 | 1.26342 | 0.02015 | 0.530864 |
| XM_001926063.5 | 100157318 | LOC100157318 | 2163 | 3.36288 | 1.36885 | 1.29673 | 0.0017 | 0.115319 |
| XM_003131161.2 | 100514726 | ENPP7 | 2143 | 0.880943 | 0.356705 | 1.30432 | 0.03125 | 0.660837 |
| XM_001928603.2 | 100157947 | NUGGC | 2936 | 4.15897 | 1.67701 | 1.31034 | 0.001 | 0.0796319 |
| XM_003358091.2 | 100621753 | PHOSPHO1 | 1872 | 2.77886 | 1.09671 | 1.3413 | 0.0307 | 0.6552 |
| XM_005655946.1 | 102158679 | LOC102158679 | 648 | 5.56455 | 2.18159 | 1.35088 | 0.0093 | 0.346515 |
| XM_005660100.1 | 102164443 | CER1 | 429 | 7.82554 | 3.05111 | 1.35886 | 0.02775 | 0.624765 |
| XM_005668016.1 | 100511582 | SYT2 | 2253 | 3.31218 | 1.27368 | 1.37877 | 0.00065 | 0.0580178 |
| XM_005672261.1 | 553951 | CCL20 | 633 | 21.7227 | 8.34418 | 1.38036 | 0.0026 | 0.154714 |
| XM_003123159.3 | 100511550 | C2H19orf59 | 1165 | 1.6688 | 0.636888 | 1.3897 | 0.0198 | 0.527357 |
| XM_001924824.2 | 100157645 | TAAR1 | 1029 | 1.36352 | 0.516483 | 1.40054 | 0.03335 | 0.681438 |
| XM_005654032.1 | 100738454 | LOC100738454 | 1683 | 2.27455 | 0.861127 | 1.40129 | 0.0043 | 0.214814 |
| XM_003357321.2 | 100623094 | TREH | 1834 | 46.6525 | 17.0326 | 1.45365 | 5.00E-05 | 0.00737594 |
| XM_005672658.1 | 102165171 | LOC102165171 | 1427 | 4.66587 | 1.59579 | 1.54787 | 0.00075 | 0.0641814 |
| XM_005658205.1 | 100620987 | LOC100620987 | 1130 | 17.7787 | 5.86977 | 1.59877 | 0.0001 | 0.0134312 |
| XM_003123966.2 | 100518899 | LECT2 | 971 | 6.8043 | 2.09781 | 1.69756 | 0.00055 | 0.0514701 |
| XM_005665463.1 | 100511937 | PIK3R3 | 4133 | 1.99538 | 0.608412 | 1.71354 | 0.01835 | 0.507325 |
| XM_003356418.3 | 100523254 | CTBS | 728 | 4.6778 | 1.37206 | 1.76949 | 0.00275 | 0.160458 |
| NM_001244729.1 | 100512958 | C3H2orf40 | 743 | 7.51321 | 2.20141 | 1.771 | 0.00065 | 0.0580178 |
| XM_001929470.3 | 100157565 | VPREB3 | 928 | 2.55062 | 0.715911 | 1.83299 | 0.00565 | 0.255305 |
| NM_001136512.1 | 100192320 | BCMO1 | 733 | 18.9554 | 5.22397 | 1.85939 | 5.00E-05 | 0.00737594 |
| NM_213978.1 | 397033 | B2M | 514 | 125.711 | 34.3076 | 1.87351 | 0.00015 | 0.0185889 |
| XM_003480520.2 | 100738878 | TMEM261 | 715 | 2.10429 | 0.511248 | 2.04124 | 0.0331 | 0.680272 |
| XM_005662419.1 | 100240745 | REG3G | 801 | 2108.04 | 510.115 | 2.04701 | 0.0053 | 0.244904 |
| XM_003123375.2 | 100516246 | LOC100516246 | 533 | 1.90404 | 0.460013 | 2.04932 | 0.02865 | 0.632903 |
| XM_005662048.1 | 397669 | CD19 | 2328 | 2.23053 | 0.511906 | 2.12344 | 0.00655 | 0.280004 |
| NM_001243919.1 | 100511955 | CUEDC1 | 2753 | 1.50056 | 0.327296 | 2.19684 | 0.0184 | 0.507613 |
| NM_213783.2 | 396674 | RETN | 520 | 4.96396 | 0.950262 | 2.38509 | 0.00795 | 0.317074 |
| XM_003129101.1 | 100524265 | CXCL13 | 1170 | 15.0795 | 2.61572 | 2.52731 | 5.00E-05 | 0.00737594 |
| XM_005661698.1 | 100518542 | LOC100518542 | 1088 | 1.27687 | 0.190614 | 2.74388 | 0.0271 | 0.616628 |
| NM_214362.1 | 397649 | LTF | 2313 | 15.9872 | 2.23348 | 2.83955 | 5.00E-05 | 0.00737594 |
| NM_001101030.1 | 100113424 | BPIFB2 | 1705 | 1.72785 | 0.239105 | 2.85326 | 0.00105 | 0.0821294 |
| XM_001928143.1 | 100156183 | ELL3 | 1751 | 3.92701 | 0.504075 | 2.96172 | 5.00E-05 | 0.00737594 |
| XM_003357425.2 | 100623585 | CR2 | 4124 | 6.09872 | 0.758916 | 3.0065 | 5.00E-05 | 0.00737594 |
| XM_003354408.2 | 100626247 | LOC100626247 | 690 | 4.68423 | 0.445272 | 3.39505 | 0.0056 | 0.253696 |
| XM_005660249.1 | 100520167 | PAX5 | 2347 | 2.27619 | 0.146993 | 3.9528 | 5.00E-05 | 0.00737594 |
| XM_005667555.1 | 100623585 | CR2 | 5252 | 3.00809 | 0.0828739 | 5.18179 | 0.0025 | 0.150671 |
| NM_214215.2 | 397423 | FABP6 | 516 | 13.0743 | 0.249872 | 5.7094 | 0.0483 | 0.795661 |
| XM_003128341.2 | 100524813 | DAXX | 2502 | 1.09892 | 0.00124275 | 9.78833 | 0.0322 | 0.671463 |
| NM_213754.2 | 396596 | LOC396596 | 737 | 0.6767 | 0 | inf | 5.00E-05 | 0.00737594 |
| XM_003355214.3 | 100523961 | LOC100523961 | 2200 | 1.29051 | 0 | inf | 3.70E-04 | 0.005842 |
