# Supplementary material for: Regulation and Molecular Mechanism of TLR5 on Resistance to Escherichia coli F18 in Weaned Piglets
Source: Animals (Basel). 2019 Sep 27;9(10):735. doi: 10.3390/ani9100735 (PMC6827021; doi:10.3390/ani9100735)
Supplement: Supplementary file 1 [file animals-09-00735-s001.zip › Figure S1.doc]

**Figure S1.** Volcano plot of differentially expressed genes (DEGs) between *E. coli* F18-resistant and -sensitive groups. The red dots indicate the upregulated DEGs; the green dots indicate downregulated DEGs; the x-axis represents the log_2_ (fold-change) value; the y-axis represents the −log_10_ (*P*-value) value; the fold-change is identified as the ratio *E. coli* F18-resistant group (SR)/sensitive group (SS).
