# Supplementary material for: Regulation and Molecular Mechanism of TLR5 on Resistance to Escherichia coli F18 in Weaned Piglets
Source: Animals (Basel). 2019 Sep 27;9(10):735. doi: 10.3390/ani9100735 (PMC6827021; doi:10.3390/ani9100735)
Supplement: Supplementary file 1 [file animals-09-00735-s001.zip › Figure S2.doc]

**Figure S2.** Histogram of GO enrichment. The x-axis shows GO classification on the second level including biological process (BP), cellular component (CC) and molecular function (MF); the y-axis shows the number of genes enriched.
