# Supplementary material for: Regulation and Molecular Mechanism of TLR5 on Resistance to Escherichia coli F18 in Weaned Piglets
Source: Animals (Basel). 2019 Sep 27;9(10):735. doi: 10.3390/ani9100735 (PMC6827021; doi:10.3390/ani9100735)
Supplement: Supplementary file 1 [file animals-09-00735-s001.zip › Figure S3.doc]

**Figure S3.** KEGG pathway analysis for differentially expressed genes. The x-axis shows enrichment factor; the y-axis shows pathway name. The size of a point represents the number of DEGs and the color of the point represents the variation range of the q-value.
