# Supplementary material for: Regulation and Molecular Mechanism of TLR5 on Resistance to Escherichia coli F18 in Weaned Piglets
Source: Animals (Basel). 2019 Sep 27;9(10):735. doi: 10.3390/ani9100735 (PMC6827021; doi:10.3390/ani9100735)
Supplement: Supplementary file 1 [file animals-09-00735-s001.zip › Figure S4.doc]

**Figure S4.** The standard curves and regression equations of five proinflammatory factors, TNF-α, IL-6, IL-8, IL-10, and IL-12.
