# Supplementary material for: Regulation and Molecular Mechanism of TLR5 on Resistance to Escherichia coli F18 in Weaned Piglets
Source: Animals (Basel). 2019 Sep 27;9(10):735. doi: 10.3390/ani9100735 (PMC6827021; doi:10.3390/ani9100735)
Supplement: Supplementary file 1 [file animals-09-00735-s001.zip › Figure S5.doc]

**Figure S5.** Amplification results of promoter and methylation sequences. A represents the amplification result of promoter fragments. B represents the digestion result of recombinant plasmid. C represents amplification results of methylation sequences, in which 1–3 represent amplification of CpG1 and 4–6 represent amplification of CpG2. M stands for Marker D2000.
