# Supplementary material for: Regulation and Molecular Mechanism of TLR5 on Resistance to Escherichia coli F18 in Weaned Piglets
Source: Animals (Basel). 2019 Sep 27;9(10):735. doi: 10.3390/ani9100735 (PMC6827021; doi:10.3390/ani9100735)
Supplement: Supplementary file 1 [file animals-09-00735-s001.zip › Table S1.doc]

**Table S1 Primer information**

| Name | Sequence of the primer | Length of products (bp) | Annealing temperature |
| --- | --- | --- | --- |
| TLR5 (qPCR) | F: 5'-GGTTCTCGCCCACCACATTA-3'  R: 5'-GGGTCCCAAAGAGTCGGAAG-3' | 123 | 60 °C |
| MyD88  (qPCR) | F: 5'-GTGCCGTCGGATGGTAGT-3'  R: 5'-CAGTGATGAACCGCAGGAT-3' | 173 | 60 °C |
| TNF-α  (qPCR) | F: 5'-CGACTCAGTGCCGAGATCAA-3'  R: 5'-CCTGCCCAGATTCAGCAAAG-3' | 158 | 60 °C |
| GAPDH (qPCR) | F: 5'-ACATCATCCCTGCTTCTACTGG-3'  R: 5'-CTCGGACGCCTGCTTCAC-3' | 188 | 60 °C |
| TLR5-P1 | F: 5'-GGACTAGTGGTGGGGGTGGGGTTCTGCTTC-3'  R: 5'-CATGCCATGGTCCTCCCCGCCACCGAAGTAG-3' | 2,000 | 61 °C |
| TLR5-P2 | F: 5'-GGACTAGTGCAAGGCATTGTTTCCTGTCAGG-3'  R: 5'-CATGCCATGGTCCTCCCCGCCACCGAAGTAG-3' | 1,500 | 59 °C |
| TLR5-P3 | F: 5'-GGACTAGTGTTTCACCCCAGAGCGCCAATCCA-3'  R: 5'-CATGCCATGGTCCTCCCCGCCACCGAAGTAG-3' | 1,000 | 66 °C |
| TLR5-P4 | F: 5'-GGACTAGTATCGTGCTCATAGGACAGAAACG-3'  R: 5'-CATGCCATGGTCCTCCCCGCCACCGAAGTAG-3' | 750 | 62 °C |
| TLR5-P5 | F: 5'-GGACTAGTGATCTCCACGGACATTTG-3'  R: 5'-CATGCCATGGTCCTCCCCGCCACCGAAGTAG-3' | 500 | 60 °C |
| TLR5-P6 | F: 5'-GGACTAGTGGCGCCTCAGAGGAGCCTCAGGG-3'  R: 5'-CATGCCATGGTCCTCCCCGCCACCGAAGTAG-3' | 200 | 60 °C |
| TLR5-P7 | F: 5'-GGACTAGTAGAGAAAAAGAAAGGGTTACCTG-3'  R: 5'-CATGCCATGGTCCTCCCCGCCACCGAAGTAG-3' | 100 | 60 °C |
| TLR5-B1 | F: 5'-AAAAGAGAAGGTAGGGTAGGATTAT-3'  R: 5'-CTCAAACTAAAATCTATCTAAATTCTAAAA-3' | 157 | 56 °C |
| TLR5-B2 | F: 5'-AAATGTTGGTTTGGATGATTTTTA-3'  R: 5'-AAACTACCTCCTACTCCAACCCTAA-3' | 258 | 60 °C |

Note: P1–P7 represent the amplification primers for different sections of the promoter region; B1 and B2 represent BSP primers for promoter methylation detection. Italics letters in P1–P7 indicate recognition sequences and protection bases of restriction endonuclease *Spe I* and *Nco I*.
